# Celibacy

**Published:** 1877-11

**Authors:** 


### CELIBACY

Is the ruin of any nation; it is a greater moral curse than drunkenness ever has been; and the parent who countenances it is, to that extent, his child's worst enemy, whether that child be son or daughter. As nations and communities prosper and grow older, the lines between wealth and poverty become more distinct; and, with equal pace, the strife for riches becomes a passion and a desperation, with a declining morality: and with all this, fewer persons marry, and they, at a later age in life—the unmarried setting up private establishments; while the more successful inaugurate a style of living largely beyond the profits of their business; when on any financial jar their rickety plans are deranged, and they are the first to go into bankruptcy. But, besides these two classes, there is a third, far outnumbering both, whose profligacy is of the more common sort, spreading its demoralizing and corrupting influences far and wide. Look at Stockholm, the capital of Sweden, once the fear of nations, when Charles the Fifth led her armies! But now, enervated by her demoralizations, she exists only by sufferance. In Stockholm, very near *half* of the registered births are illegitimate. In Paris, one child in every three is born out of wedlock. It is very easy to see that the cause of this is largely owing to deferred marriage. In our own country, especially in our large cities, the fact is everywhere observed, that young men are putting off marriage till a later and later period of life. Very many do not entertain the thought of it until past thirty.

But why do young men put off marriage until thirty and over? The reasons are twofold. Poverty and pride. They cannot afford to live in the style which their ambition aspires to. Young people want to begin at too high a round in the social ladder. Formerly, a young man was respected who married, and lived in the rear of his house, while the front was his office, or shop, or store; and when he got a little farther on, he occupied the first floor as his place of business, while his wife and children lived up stairs, *and it ought to be so still*, by three-fourths of the business men of our large cities. It is capable of demonstration, that it is healthier to do so. and a great economizer of time and money. To live "*up town*," for example, in New York, three or four miles away from a man's place of business, is neither as good for himself or family, soul, body, or estate, *in the long run*, as if he did business on the first floor, and his family lived on the second or third of the same building.

A business man is always anxious when away from his place of business. He has a peace of mind when on the spot which is of itself a treasure. Bear us witness of this ye business men, who live in streets above the thirties, or "*out of town*."

By the old plan, men enjoyed their families, lived with their wives and children, ate at home always, always slept at home; and in the end, lived longer, happier, and died richer, and more honored, than do the same class of persons now-a-days, with their empty store-lofts down town, and their dwellings miles away, at an extra rent of from one to three thousand dollars a year.

Compare the hasty and unmasticated dinner, or the late repast of five P. M., all jaded, exhausted, depressed with ten hours' labor, with the cozy dinner at decent old-fashioned noon—just hungry enough to enjoy, just tired enough to make a half-hours' sitting grateful, and just exhilarated enough with the profits of the morning to make it a matter of pleasureable converse while eating.

